# Understanding Hospital Admissions Close to the End of Life (ACE) Study

**DOI:** 10.1186/1472-6963-13-89

**Published:** 2013-03-11

**Authors:** Zoë Slote Morris, Miranda Fyfe, Natalie Momen, Sarah Hoare, Stephen Barclay

**Affiliations:** 1 Institute of Public Health, University of Cambridge, Forvie Site, Robinson Way, Cambridge, CB2 0SR, UK; 2 Formerly of NHS Cambridgeshire, 2nd Floor, Lockton House, Clarendon Road, Cambridge, CB2 8FH, UK; 3 Primary Care Unit, Department of Public Health and Primary Care, University of Cambridge, Forvie Site, Robinson Way, Cambridge, CB2 0SR, UK

**Keywords:** End-of-life care, Palliative care, Place of death, Inappropriate admissions, Mixed methods, Vignettes, Framework analysis, Social marketing, Behaviour change, Ethics

## Abstract

**Background:**

Palliative care is a policy priority internationally. In England, policymakers are seeking to develop high quality care for all by focusing on reducing the number of patients who die in acute hospitals. It is argued that reducing ‘inappropriate’ hospital admissions will lead to an improvement in the quality of care and provide cost savings.

Yet what is meant by an ‘inappropriate’ admission is unclear and is unlikely to be shared by all stakeholders. The decision process that leads to hospital admission is often challenging, particularly when patients are frail and elderly. The ACE study reopens the idea of ‘inappropriate’ hospital admissions close to the end of life. We will explore how decisions that result in inpatient admissions close to death are made and valued from the perspective of the decision-maker, and will consider the implications of these findings for current policy and practice.

**Design/Methods:**

The study focuses on the admission of patients with advanced dementia, chest disease or cancer who die within 72 hours of admission to acute hospitals. The study uses mixed methods with three data collection phases. Phase one involves patient case studies of admissions with interviews with clinicians involved in the admission and next-of-kin. Phase two uses vignette-based focus groups with clinical professionals and patients living with the conditions of interest. Phase three uses questionnaires distributed to clinical stakeholders. Qualitative data will be explored using framework analysis whilst the questionnaire data will be examined using descriptive statistical analysis. Findings will be used to evaluate current policy and literature.

**Discussion:**

Significant ethical and validity issues arise due to the retrospective nature of phase one of the study. We are not able to gain consent from patients who have died, and the views of the deceased patients cannot be included directly, which risks privileging professional views. This phase also relies on the memories of the participants which may be unreliable. Later phases of the study attempt to compensate for the “absent voices” of the deceased patients by including next-of-kin and patient focus groups.

## Background

Policymakers in many Western countries are attempting to improve the quality of Palliative care and have developed strategies to help them do this [1-4]. The aim of the English *End of Life Care Strategy* for example, “is to bring about a step change in access to high quality care for all people approaching the end of life… High quality care should be available wherever the person may be: at home, in a care home, in hospital, in a hospice or elsewhere” [4]. Health services must attempt to improve care within the context of ageing populations, increasing death rates and economic constraints.

Despite policy rhetoric around improving *quality* of care independent of place, the dominant policy discourse around the *problem* of Palliative care centres upon place. Acute settings are also seen as unpleasant places in which to die [5,6], and it is argued that, as most people express a wish to die at home, patient choice should be met through reducing the rate of inpatient deaths. There is a belief also that Palliative care competes unnecessarily for scarce resources and could be made more cost-effective if the rates of death in acute hospitals could be reduced in favour of deaths at home [7-9].

Policymakers are hopeful that with better access to 24-hour community care and greater staff awareness, the number of inappropriate admissions will reduce – with cost-savings following.

There are, however, a number of issues which challenge the validity of these assumptions and therefore could influence the success of the policy. One is the assumption that patients and carers will respond to greater and improved opportunities for home care by making decisions which avoid hospital admissions. How decisions that lead to admissions at the very end of life are actually made in practice is largely unknown. However, retrospective studies suggest that recognition of a patient being near the end of life often comes late, justifications for admission (or not) are not shared amongst stakeholders [10-13], and that clinician and informal caregiver behaviour, and therefore behaviour change, is guided by a complex range of factors which go beyond training to support technical efficiency, including emotion, habit, belief, and so forth [14-16]. The literature also suggests that many patients do not have a preference, or change their preferences away from home to hospital as their illness progresses [17,18]. Often, a hospital admission is not seen as inappropriate to a patient or carer, but more an inevitability, even if many patients do aspire to die at home [19-21]. Questions about the assumptions of policy have implications for both policy and practice.

Mismatches between policy assumptions and those of frontline carers may influence the implementation of current policy (reducing admissions) as people are unlikely to respond as intended. Mismatches also question the appropriateness of current policy solutions to the wider policy aim of increasing quality of care and enabling choice of place at the end of life. For these reasons, the ACE Study seeks to reopen the question of “inappropriate” or “avoidable” admissions close to the end of life. It aims to understand the decision processes that result in a patient being admitted to hospital where they die soon afterwards. It is designed to explore how decisions resulting in inpatient admissions close to death are made and valued from the perspective of the decision-makers themselves, and to consider the implications for policy and practice.

[Table 1. Details the research questions, study objectives and analysis].

**Table 1:** How the research questions will be addressed by data collection methods and analysis

**Research questions**	**Data collection**	**Data analysis**
1. How are decisions that result in admissions at end of life made in practice?	• Qualitative interviews with the decision-makers in the community and hospital and with next of kin of 48 patients will be used to develop descriptive case-studies of patient experiences	• Thematic analysis of interview data
		• Quantitative analysis of questionnaire
2. What do patients, carers and practitioners think can or should be different in decision-making around admissions to hospital close to the end of life?	• Vignette-based focus groups with patients/carers and commissioners/managers to explore their perspectives on acute admissions at end of life	• Thematic analysis of interview data
		• Quantitative analysis of questionnaire
	• Qualitative vignette-style questionnaires with professionals to validate and quantify findings from the interview phase	• Thematic analysis of patient focus groups
3. What is current policy around place of death and hospital admissions close to the end of life?	• Review and evaluation of literature and policy against the empirical findings	• Thematic analysis of literature and policy
		• Thematic analysis of commissioner/manager focus groups
4. How do current policy and practice compare?	• Review and evaluation of literature and policy against the empirical findings	• Critical comparison of thematic analysis of empirical data and thematic analysis of policy to help identify overlaps, gaps, contradictions and tensions.
5. What are the implications for policy and practice?	• Review and evaluation of literature and policy against the empirical findings	• Application of social marketing framework to help identify “actionable insights” for policy and practice.

## Methods and Design

### Design

The study uses mixed methods. Qualitative data will help describe how admissions are understood and viewed from “the ground up”. Quantitative data will help determine the extent of the attitudes, perspectives and intentions we have gathered from the qualitative part of the study reflect patient and practitioners experiences. Together, these data will then be used to evaluate current services and inform policy.

### Setting

The study focuses on two adjoining but separate administrative areas, with different demographic profiles relevant to place of death, including access to services. The first area is largely rural, with a university city and larger towns and areas of significant social deprivation and affluence. The second area is mainly urban, characterised by high levels of ethnic diversity and significant pockets of urban deprivation. The study area includes one large Primary Care Trust (PCT) which will shortly become a Clinical Commissioning Group (CCG), responsible for purchasing care for the local communities. There are two providers of Out of Hours (OOH) primary care and two Local Authorities responsible for social care. The study will focus on the two acute settings in the study areas.

### Data collection

The study is organised in three data collection phases, encompassing five research tasks.

### Phase 1: Patient case-studies

**Figure 1:**
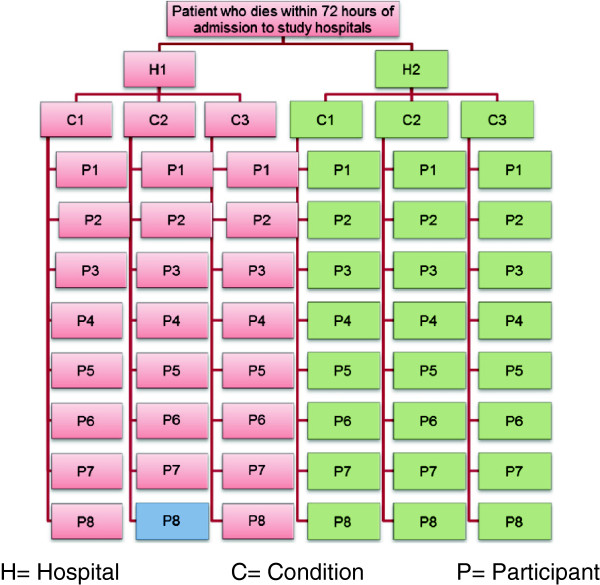
Participant structure of the study.

Phase 1 of the study adopts a qualitative case-study approach centred on patients aged 65 or older with advanced dementia, Chronic Obstructive Pulmonary Disease, or cancer who die within 72 hours of admission to of the two hospitals. Patients will be identified retrospectively with help from the hospitals’ Bereavement Services. These Services receive the hospital notes and organise the death certificates and other paperwork that needs to be completed following all deaths in hospital: the notes will be used to identify patients for the case studies. The Services also provide comprehensive bereavement support or onward referral as appropriate. The study aims to identify a maximum of 48 patient case-studies, half from each hospital, with approximately eight patients in each condition group. This is shown diagrammatically in Figure 1. Patients will be purposively sampled to ensure coverage of a range of variables known to predict place of death [4,22].

**Figure 2:**
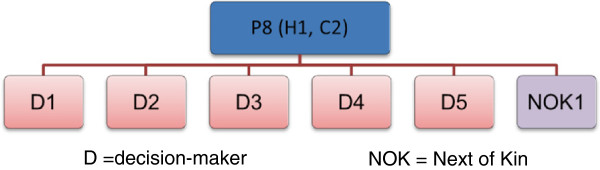
How participants relate to the deceased patient.

Participants in Phase 1 are (1) community and hospital staff involved in the admission who will be interviewed shortly after the death and (2) patients’ next of kin, who will be interviewed three to six months after the patient died. Up to six people involved with each patient’s care will be invited for interview, including one next of kin if possible. Staff will be identified from patient medical records and then through a “chain” or snowball sampling (See Figure 2).

The recorded next of kin in the deceased’s medical record will be contacted by the hospitals’ bereavement offices and invited to take part in the study. Participants will be asked to return a reply-slip indicating interest to the university-based research team. In both cases, data will be collected through semi-structured interviews.

The interviews are designed to explore carers’ descriptions and explanations of what happened and why; the factors that influenced the decision to admit the patient to hospital; how they view the decision in hindsight and what, if anything in their view, could or should have been done differently to prevent admission. Interviews with the next of kin will cover similar issues as well participant’s thoughts on what the patient wanted or might have wanted; and what, if anything, they would like to have been different in the support given.

### Phase 2: Non-clinical stakeholder perspectives

Phase 2 of the study involves vignette-based focus groups with patients and carers living with the three conditions of interest to gain a patient perspective. Focus groups will be organised by place and condition (n=6 groups of 6–8 participants). Participants will be identified through hospital services, support groups, the ACE Study user group and existing patient and public involvement networks. Interested participants who reply to the research team will be invited to take part in the study if they meet eligibility criteria.

The focus group will involve the researchers telling a brief vignette story about a patient admitted to hospital who dies shortly afterwards and, depending on time, a patient who dies at home. Participants will be asked to comment on issues such as: what the story tells them; what they might expect to happen; what the people in the story want to happen; what they expect from taking the patient to hospital; what reasons there are for not taking the patient to hospital; what the right thing to do was; what alternative options there were.

A qualitative vignette approach was chosen as being particularly well suited to supporting discussion around topics that are “difficult to broach” or potentially upsetting [23], by creating distance between the participant and the topic [24]. This approach is similar to that employed by Gott et al. in their study of older people's preferences for place of care at end of life [19].

We will use the same vignette approach with a group of senior policymakers and commissioners to compare responses. Participants will be identified through existing professional networks and snowball sampling.

### Phase 3: Clinical stakeholder perspectives

In Phase 3, a vignette-style questionnaire for staff in hospitals and the community will validate and quantify earlier findings. The research team will compile a database of relevant professionals within the National Health Service (NHS) boundary of the area. This will include GPs, District Nurses, OOH staff, ambulance staff; palliative care specialists in hospitals and the community, and hospital staff who care for older people, in particular Registrars and Consultants in Geriatrics, Chest Medicine, Oncology, and Emergency Medicine. District Nurses will be identified though area managers, and hospital staff through a “snowball” technique working with departments and existing research team contacts. Details of GPs are in the public domain. A questionnaire will be sent to everyone on the database and it is anticipated that the sample size will be approximately 1000.

The focus of the questionnaire will include a set of patient vignettes with closed multiple-choice options about whether the patient should be admitted or referred, followed by open text boxes asking for reasons and comments. These vignettes will be developed from case-studies in the interview phase of the research, with further input from clinicians and the user group. Vignette questionnaires are considered superior to a traditional questionnaire format because the questions asked are less abstract and closer to real-life [25]. When used quantitatively they also allow the collection of data from large numbers of people [24,26] and are particularly good for exploring ill-defined issues or dilemmas [27].

### Analysis

Table 1 summarises how the data will be analysed to answer the research questions. Data analysis will happen in two phases. First the empirical data will be analysed. Secondly, the findings will be used to evaluate current policy around palliative care and make policy recommendations.

### Analysis of empirical data

Data from interviews will be organised thematically using an adapted framework analysis approach [28]. This uses a thematic template [29] and a data recording matrix of theme against participant to identify and organise responses for further analysis and interpretation [30]. The focus of the analysis will be to ascertain participant’s perspective, including their opinions, values and assumptions. This data is likely to be gathered from indirect questioning about the participants experience.

**Figure 3:**
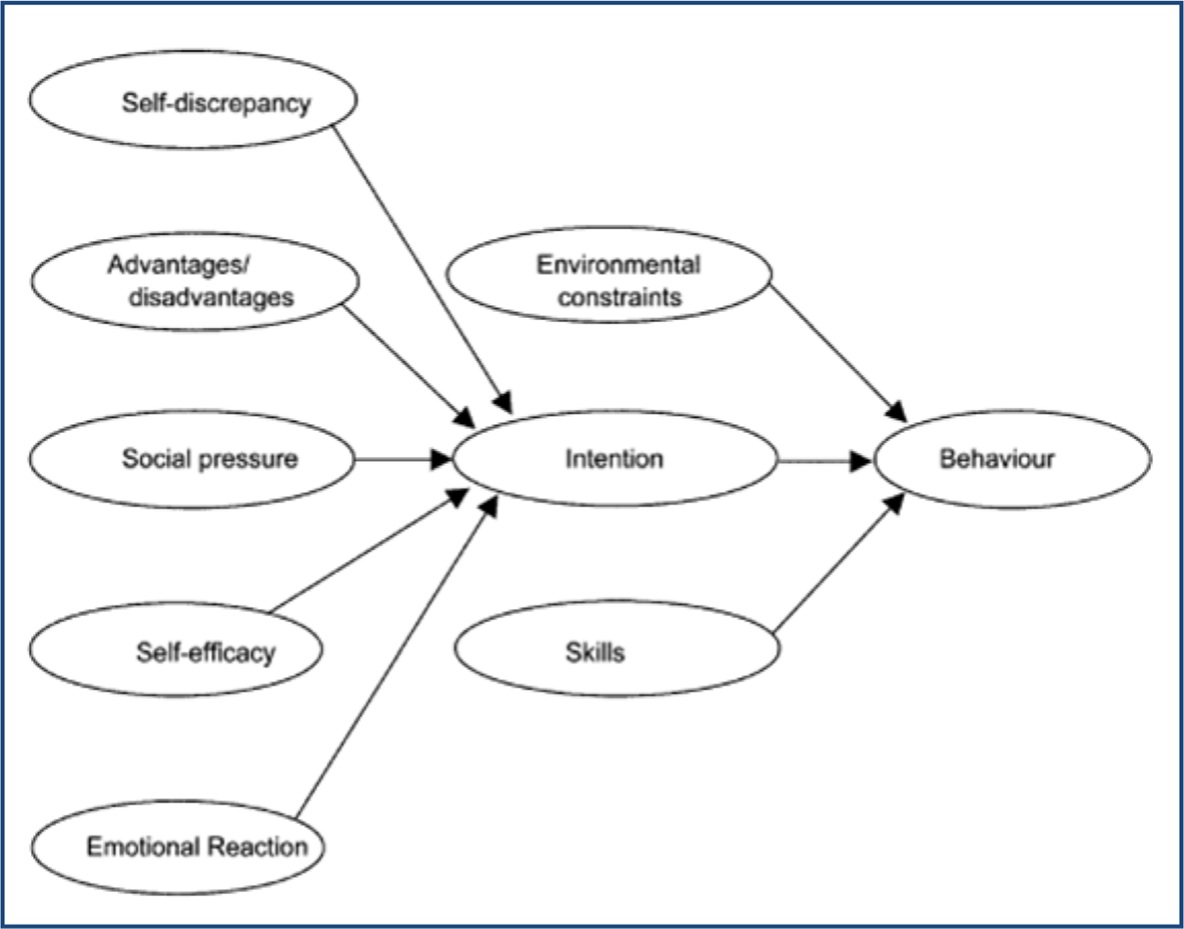
**A simplified model of behaviour **[14]**.**

The analysis approach will be guided by psychological models of behaviour and will use a social marketing framework to help highlight key factors in decision-making. Psychological models of behaviour change are useful for understanding behaviour as they can help direct analytical attention to issues that might be relevant to behaviour change [14,15] and are well established in health psychology. Social cognition theories relate to how individuals make sense of social situations, recognising that individual behaviour is influenced by a number of factors such as a person’s belief and their self-efficacy. An example of these models of behaviour is shown in Figure 3.

Social marketing is used in public health to help bring about behaviour change [31]. It starts with generating “insights” into actors’ current practices, beliefs, and preferences which influence their behavioural choices. It assumes that individual behaviour is premised on many factors with trade-offs between them, and that current practice may have benefits which are forgone if the behaviour is changed, such as emotional well-being and social acceptance. In Public Health research these insights have been used to develop “attractive exchanges” [32], designed to support the adoption of new behaviours.

Social marketing assists analysis by focusing attention on the factors that influence behaviour from the perspective of the actors themselves, without attempting to attribute blame. The current palliative care policy directed towards reducing inpatient death does not explore why formal and informal caregivers decide to admit a patient and using an analysis approach that is focused on behaviour will provide useful insight. Applying the social marketing framework to the data will help order the analysis and generate future policy options for providing high quality Palliative care grounded in the empirical data.

The questionnaire data will be analysed quantitatively, requiring open questions to be coded. The sampling frame will be the entire population of healthcare professionals involved in decision concerning hospital admissions in the two hospitals studied and the surrounding community teams. We estimate this to be around 1000 professionals. The questionnaire is not designed for hypothesis testing and will generate primarily descriptive data. Basic descriptive analysis of categorical sets such as professional group or geographical area will be undertaken in SPSS, using methods including chi-square, logistical regression and cluster analysis as appropriate. Analysis categories will be derived from the literature and cluster analysis. Questionnaire results will be collated with the main qualitative analysis and used to evaluate current policy and make recommendations.

The second stage of the analysis will evaluate current policy and literature in the light of the empirical findings. We expect that combining the behaviour model and social marketing framework will provide powerful analytical leverage to offer a clear description of current behaviour and its associates, and a transparent means to evaluate current policy. This analysis is intended to be solution-focused; to provide “actionable insights” to inform effective policy.

### Ethical issues

Palliative care is recognised as a sensitive area to research. As such, a considerable time has been spent discussing the protocol and instruments with clinician stakeholders and the user group, who have advised on feasibility and acceptability. A central issue was the identification of suitable patients and the individuals who had been involved in the decision which had led to their admission to hospital. Consent could not be gained from the deceased patient and neither was proxy consent from next of kin possible. This was both because a patient's notes remain confidential to the patient after death and because our user group advised against contacting bereaved relatives so soon after death. Instead we gained approval from the National Information Governance Board (NIGB) to view deceased patients’ records to collect relevant information and identify an initial contact. In addition we have collaborated with the hospital bereavement services who will initially approach next of kin and invite them to participate in the study.

Working with the bereavement services is beneficial to maintaining the confidentiality of participants. In particular, the invitation letter to the patient’s next of kin will come from the care provider, thus ensuring patient and personal data remains within the service. In turn, the service will not know who responded to the research team, which will help increase the anonymity of the responses.

It is usual when undertaking research with people who have been bereaved or who are very ill to inform participants that you will contact their GP on their behalf if they become worryingly distressed during the interview and refuse to seek help themselves. In this case, the participants are not known to the researchers as patients, so it will be necessary to gather information about the participants’ GP before they are interviewed. The research ethics committee reviewing the study also requested that the interviewer follow up next of kin participants a couple of days after interview to check on their well-being. In addition we were asked to prepare an information sheet on sources of bereavement support to leave with participants.

The topic of care at the end of life is potentially very emotionally charged for all participants: care professionals who may have regrets over decisions made, and informal carers who are recently bereaved. However, since care in last few days of life is the focus of the study, we have been explicit in our material to ensure that participants are not surprised by this topic being raised. While this may bias the sample, this approach we believe is more ethical and is unlikely to be detrimental to overall validity of the study.

The vignette design of the focus groups will allow participants to distance themselves from the topic if they wish. The focus groups with patients and carers will be attended by two researchers, so that one can support any participant should they become distressed. At their own suggestion, members of the project's user group have volunteered to attend the focus groups to help support and reassure participants. At the end of the focus group participants will be debriefed and asked if they have any concerns arising from the group session. They will be signposted to contacts from where they could gain support. We will seek to arrange for an appropriate specialist nurse to come at the end of the group session to take any questions and help debrief participants.

### Approvals

The study is approved by the Hertfordshire Research Ethics Committee (England) (#11/EE/0491), and National Information Governance Board (NIGB) (ECC 1–05 (g)/2012).

## Discussion

The ACE Study faces a number of challenges.

1) There are ethical and data protection issues related to the impossibility of obtaining patient consent and the sensitive nature of the topic. Resolving these has added layers of research ethics and governance processes and delayed the study by about six months. This has been very challenging as the study has a finite end date due to funding.

2) The retrospective design of Phase 1 necessarily excludes patients and may therefore privilege professional views. We have attempted to compensate for this by including bereaved carers/family, as recipients of care themselves as well as patient proxies. Whilst imperfect, this is a commonly used strategy in palliative care research [33]. In addition, retrospective studies using proxies can illuminate the experiences of people who died but were not previously identified as “end of life” and those who would have been too ill to take part themselves [34].

3) Including next of kin in the design presents additional ethical challenges. If attempts are made to protect participants, and bereaved relatives in particular, they are excluded and patronised [35]. There is a balance between autonomy and non-maleficence [36,37]. The ACE Study has attempted to include people and offer support without assuming they cannot cope, in which we are guided by existing models of acceptable practice [38].

4) The absent voice of the patient is brought in through focus groups with patient and carer groups in Phase 2. Vignettes will be used to place distance between the participants’ personal experience and the discussion [19]. Tang’s study of preferences of people with dementia and their carers show that the need to make choices about care at end of life is one of the strongest sources of emotional distress [39]. The vignettes will help participants through this without reference to *their* choice.

5) For some there will also be concerns about the validity of the data, particularly in the first phase which is retrospective and asks participants to recall what happened. It can be argued that memories are unreliable sources of description of what actually happened, made more insubstantial by reinterpretation through hindsight and social desirability. We will attempt to speak to professionals as soon after the death as possible (aiming for within two weeks) to assist recall. However, the process of interviewing can also be viewed as an essential element of sense-making, reflection and learning [40] through the development of “narratives” [41] Interpretation, reflection and learning are more likely to guide future action than forgotten “truths”. Their accounts will also reveal the context of the decision-making process. Participants will articulate what they considered pertinent in the decision, and thus will reveal attitudes and values that inform decision-making at the end of life.

Despite these limitations, the ACE Study will provide a unique descriptive account of the decision-pathway that ends with an inpatient death soon after admission, how these are understood and valued by stakeholders, and what this means for policy and practice. A summary of how the study will add to existing knowledge is presented below.

The ultimate aim is to provide knowledge that can be used to improve patient care. Better insight into the processes and reasons for current decisions concerning hospital admissions close to death can support better targeting of effort to improve the quality of palliative care. These questions and themes are relevant to policymakers and practitioners around the world, many of whom are currently seeking to reduce hospital admissions close to the end of life and increase the number of deaths that occur at home, while at the same time as enabling choice and “a good death”. The ACE Study will help confirm whether these aspirations align or not, and highlight some future options for policy and practice.

### Summary of how the study will add to existing knowledge

What the study will add

•Understanding of the end of life care decision-making for the patients with non-malignant as well as malignant diseases.

•Understanding of decision-making “in context” and around an actual critical event, rather than a hypothetical (improved ecological validity).

•Multiple perspectives on the same admission revealing issues relevant to the health care system, including views of informal carers and non-specialist palliative care clinicians.

•An empirically-derived model of actual decision-making about admission at the very end of life to inform policy and practice.

•An empirically-derived definition of “inappropriate” admissions.

•Applied, solution-focused approach designed to support policy and service development.

## Abbreviations

(NHS): National Health Service; (NIGB): National Information Governance Board; (REC): Research Ethics Committee; (PCT): Primary Care Trust.

## Competing interests

The authors declare that they have no competing interests.

## Authors’ contributions

ZM is the primary author of this manuscript and guarantor. All authors gave the paper critical review and were involved in revising the manuscript. All authors read and approved the final manuscript. SB is the chief investigator and ZM is the lead researcher. All authors made a substantial contribution of the development of the protocol.

## Pre-publication history

The pre-publication history for this paper can be accessed here:

http://www.biomedcentral.com/1472-6963/13/89/prepub
